# Supplementary material for: Community Health Workers as a Strategy to Tackle Psychosocial Suffering Due to Physical Distancing: A Randomized Controlled Trial
Source: Int J Environ Res Public Health. 2021 Mar 17;18(6):3097. doi: 10.3390/ijerph18063097 (PMC8002631; doi:10.3390/ijerph18063097)
Supplement: Supplementary file 1 [file ijerph-18-03097-s001.pdf]

Supplement S1 Baseline questionnaire

Corona-related questions

On a scale from 0 to 10 (where 0 means “not at all satisfied” and 10 means “completely satisfied”):

L 01. Are you afraid to get corona?

0	1	2	3	4	5	6	7	8	9	10

L 02. If a new 'stay in-your-home measure' were to be announced in winter, how bad would you find it?

0	1	2	3	4	5	6	7	8	9	10

L 03. Are you afraid for the future?

0	1	2	3	4	5	6	7	8	9	10

L 04. Are you afraid to visit your GP at this time?

0	1	2	3	4	5	6	7	8	9	10

L 05. Are you afraid to visit the dentist at this time?

0	1	2	3	4	5	6	7	8	9	10

L 06. Are you afraid to visit the hospital at this time?

0	1	2	3	4	5	6	7	8	9	10

L 07. Are you afraid to go to the store?

0	1	2	3	4	5	6	7	8	9	10

L 08. Are you afraid to get out?

0	1	2	3	4	5	6	7	8	9	10

L 09. Are you afraid to cross someone in the street?

0	1	2	3	4	5	6	7	8	9	10

Quality of life (HIS, Sciensano)

On a scale from 0 to 10 (where 0 means “not at all satisfied” and 10 means “completely satisfied”):

L 01. How much support do you get from family and friends?

0	1	2	3	4	5	6	7	8	9	10

L 02. How satisfied do you currently feel with your life as a whole?

0	1	2	3	4	5	6	7	8	9	10

Emotional support (PROMIS)

	Never	Rarely	Sometimes	Usually	Always
E 01. I have people who care about what happens to me	1	2	3	4	5
E 02. There are people I can talk to	1	2	3	4	5
E 03. I have someone who will listen to me when I need to talk	1	2	3	4	5
E 04. I have someone to confide in or talk to about myself or my problems	1	2	3	4	5
E 05. I have someone who makes me feel appreciated	1	2	3	4	5
E 06. I have someone to talk with when I have a bad day	1	2	3	4	5
E 07. I have someone I trust to talk with about my feelings	1	2	3	4	5
E 08. I can talk about the corona period.	1	2	3	4	5

Include patient in the study if E01 + E02 ≤ 7

Social isolation (PROMIS)

	Never	Rarely	Sometimes	Usually	Always
S 01. I feel left out.	1	2	3	4	5
S 02. I feel that people barely know me.	1	2	3	4	5
S 03. I feel isolated from others.	1	2	3	4	5
S 04. I feel that people are around me but not with me.	1	2	3	4	5
S 05. I feel detached from other people.	1	2	3	4	5
S 06. I feel like a stranger to those around me	1	2	3	4	5
S 07. I feel like I am no longer close to anyone	1	2	3	4	5

Ability to participate in social roles and activities (PROMIS)

	Never	Rarely	Sometimes	Usually	Always
P 01. I have trouble doing my regular daily work around the house.	1	2	3	4	5
P 02. I have trouble participating in recreational activities with others.	1	2	3	4	5
P 03. I feel limited in my ability to visit friends.	1	2	3	4	5
P 04. I have trouble doing all of the work that I feel I should do (include work at home).	1	2	3	4	5
P 05. I have trouble keeping in touch with others.	1	2	3	4	5

Anxiety (PROMIS)

In the past 7 days:

	Never	Rarely	Sometimes	Usually	Always
A 01. I felt fearful.	1	2	3	4	5
A 02. I felt uneasy.	1	2	3	4	5
A 03. I found it hard to focus on anything other than my anxiety.	1	2	3	4	5
A 04. My worries overwhelmed me.	1	2	3	4	5
A 05. Many situations made me worry.	1	2	3	4	5
A 06. I felt something awful would happen.	1	2	3	4	5

Include patient in the study if score on A01 + A02 ≥ 7

Socio-demographics

D 01. Age

D 02. Sex

1. M
2. F
3. X

D 03. Highest degree achieved

1. No or primary school
2. Primary secondary education
3. Higher secondary education
4. Higher education (university, college)

D 04. What kind of work or activity do you do?

1. Student
2. Worker/Servant/Self-employed
3. Job-seeking
4. Houseman/housewife
5. Retired
6. Other

D 05. How many people live with you under the same roof?

D 06. In what country have you been born?

D 07. How many years do you live in Belgium?

Post-intervention questionnaire

Corona-related questions

On a scale from 0 to 10 (where 0 means “completely disagree” and 10 means “completely agree”):

D 01. Are you afraid to get corona?

0	1	2	3	4	5	6	7	8	9	10

D 02. If a new 'stay in-your-home measure' were to be announced in winter, how bad would you find it?

0	1	2	3	4	5	6	7	8	9	10

D 03. Are you afraid for the future?

0	1	2	3	4	5	6	7	8	9	10

D 04. Are you afraid to visit your GP at this time?

0	1	2	3	4	5	6	7	8	9	10

D 05. Are you afraid to visit the dentist at this time?

0	1	2	3	4	5	6	7	8	9	10

D 06. Are you afraid to visit the hospital at this time?

0	1	2	3	4	5	6	7	8	9	10

D 07. Are you afraid to go to the store?

0	1	2	3	4	5	6	7	8	9	10

D 08. Are you afraid to get out?

0	1	2	3	4	5	6	7	8	9	10

D 09. Are you afraid to cross someone in the street?

0	1	2	3	4	5	6	7	8	9	10

Quality of life (HIS, Sciensano)

On a scale from 0 to 10 (where 0 means “not at all satisfied” and 10 means “completely satisfied”):

L 01. How much support do you get from family and friends?

0	1	2	3	4	5	6	7	8	9	10

L 02. How satisfied do you currently feel with your life as a whole?

0	1	2	3	4	5	6	7	8	9	10

Emotional support (PROMIS)

	Never	Rarely	Sometimes	Usually	Always
E 01. I have people who care about what happens to me	1	2	3	4	5
E 02. There are people I can talk to	1	2	3	4	5
E 03. I have someone who will listen to me when I need to talk	1	2	3	4	5
E 04. I have someone to confide in or talk to about myself or my problems	1	2	3	4	5
E 05. I have someone who makes me feel appreciated	1	2	3	4	5
E 06. I have someone to talk with when I have a bad day	1	2	3	4	5
E 07. I have someone I trust to talk with about my feelings	1	2	3	4	5
E 08. I can talk about the corona period.	1	2	3	4	5

Social isolation (PROMIS)

		Never	Rarely	Sometimes	Usually	Always
S 01.	I feel left out.	1	2	3	4	5
S 02.	I feel that people barely know me.	1	2	3	4	5
S 03.	I feel isolated from others.	1	2	3	4	5
S 04.	I feel that people are around me but not with me.	1	2	3	4	5
S 05.	I feel detached from other people.	1	2	3	4	5
S 06.	I feel like a stranger to those around me	1	2	3	4	5
S 07.	I feel like I am no longer close to anyone	1	2	3	4	5

Ability to participate in social roles and activities (PROMIS)

		Never	Rarely	Sometimes	Usually	Always
P 01.	I have trouble doing my regular daily work around the house.	1	2	3	4	5
P 02.	I have trouble participating in recreational activities with others.	1	2	3	4	5
P 03.	I feel limited in my ability to visit friends.	1	2	3	4	5
P 04.	I have trouble doing all of the work that I feel I should do (include work at home).	1	2	3	4	5
P 05.	I have trouble keeping in touch with others.	1	2	3	4	5

Anxiety (PROMIS)

In the past 7 days:

	Never	Rarely	Sometimes	Usually	Always
A 01. I felt fearful.	1	2	3	4	5
A 02. I felt uneasy.	1	2	3	4	5
A 03. I found it hard to focus on anything other than my anxiety.	1	2	3	4	5
A 04. My worries overwhelmed me.	1	2	3	4	5
A 05. Many situations made me worry.	1	2	3	4	5
A 06. I felt something awful would happen.	1	2	3	4	5

Physical function (PROMIS)

	Without any difficulty	With a little difficulty	With some difficulty	With much difficulty	Unable to do
F 01. Are you able to do chores such as vacuuming or yard work?	5	4	3	2	1
F 02. Are you able to go up and down stairs at a normal pace?	5	4	3	2	1
F 03. Are you able to go for a walk of at least 15 minutes?	5	4	3	2	1
F 04. Are you able to run errands and shop?	5	4	3	2	1

Socio-demographics

SD 01. Did you receive support from a psychologist/psychiatrist/medication in the past year because of mental illness?

1. Yes
2. No

SD 02. Do you have any of the following diseases right now?

1. Diabetes
2. Lung disease
3. Heart disease

4. Kidney disease
5. None of the above

SD 03. What kind of work or activity do you do? (Main activity)

1. Student
2. Worker/Servant/Self-employed
3. Job-seeking
4. Houseman/housewife
5. Retired
6. Disability
7. Integration course
8. Other : ………………………………………………………….

SD 04. Are you currently getting help at home through one of the following: cleaning aid, meals at home, family help, home nursing, physiotherapist?

1. Yes
2. No

SD 05. Has this help at home been partially or completely discontinued during corona?

1. Yes, has been discontinued
2. Yes, has been discontinued, but has been (partially) restarted in the meantime
3. No, hasn't been discontinued

SD 06. Can you make ends meet with your income? Which of the descriptions comes closest to your picture of your household's income? (European social survey)

1. Living comfortably with income
2. Manages to make ends meet with income
3. Hard to make ends meet with income
4. Very difficult to make ends meet with income

SD 07. Has this become more difficult since corona?

1. Yes
2. No

SD 08. How well do you speak Dutch?

1. Fluent / level mother tongue
2. Sufficient
3. Moderate
4. Bad
5. Not at all

SD 09. In which country was your mother born?

PGIC

PGIC 01. Since the last questionnaire, have you felt a change in the extent to which you receive emotional support? (By this we mean talking about things that keep you busy and someone you can trust)

-5 -4 -3 -2 -1 0 1 2 3 4 5

Very much worse | Unchanged | Very much better

PGIC 02. Since the last questionnaire, have you felt a change in the extent to which you feel isolated?

-5 -4 -3 -2 -1 0 1 2 3 4 5

Very much worse | Unchanged | Very much better

PGIC 03. Since the last questionnaire, have you felt a change in participating in your daily activities?

-5 -4 -3 -2 -1 0 1 2 3 4 5

Very much worse | Unchanged | Very much better

PGIC 04. Since the last questionnaire, have you felt a change in the extent to which you feel anxious?

-5 -4 -3 -2 -1 0 1 2 3 4 5

Very much worse | Unchanged | Very much better

PGIC 05. Since the last questionnaire, have you felt a change in your fear of corona?

-5 -4 -3 -2 -1 0 1 2 3 4 5

Very much worse | Unchanged | Very much better

Satisfaction with the intervention

Has a volunteer from our corona project contacted you in the last few weeks?

1. Yes
2. No

If yes:

On a scale from 0 to 10 (where 0 means “not at all satisfied” and 10 means “completely satisfied”):

T 01. How satisfied are you with the project?

0	1	2	3	4	5	6	7	8	9	10

T 02. How satisfied are you with the contacts with the volunteer?

0	1	2	3	4	5	6	7	8	9	10

T 03. If there would be another lockdown period in winter, would you want someone to contact you again? (Application via general practitioner in the future)

0	1	2	3	4	5	6	7	8	9	10

T 04. Would you recommend this project to someone in your own environment?

0	1	2	3	4	5	6	7	8	9	10

T 05. Are you prepared to a further conversation about your experiences over the past 6 weeks?

1. Yes
2. No
